# Single-nucleotide polymorphism rs731384 is associated with plasma lipid levels and the risk of coronary artery disease in Chinese populations

**DOI:** 10.1042/BSR20181502

**Published:** 2018-12-18

**Authors:** Chenhui Zhao, Mingyue Ji, Jing Zhang, Qiaowei Jia, Jieyin Liu, Fenghui An, Zhaohong Chen, Lihua Li, Liansheng Wang, Wenzhu Ma, Zhijian Yang, En-Zhi Jia

**Affiliations:** 1 Department of Cardiovascular Medicine, The First Affiliated Hospital of Nanjing Medical University, Nanjing 210029, Jiangsu Province, China; 2 Lianshui County People’s Hospital, Huaian, China; 3 Department of Cardiovascular Medicine, The Friendship Hospital of Ili Kazakh Autonomous Prefecture, Yining 835000, China

**Keywords:** coronary artery disease, miR-130a, Serum lipid, Single nucleotide polymorphism

## Abstract

Aims: To investigate the relationship between the miR-130a polymorphism rs731384 and coronary artery disease (CAD) and to further explore the molecular mechanism of the pathogenesis of CAD, an observational single-center study was conducted. Method: A total of 876 subjects were recruited in the present study. Four milliliters of venous blood was drawn after 12 h of fasting to perform biochemical assays. CAD patients and controls were distinguished by coronary angiography. Rs731384 was genotyped on the Agena MassARRAY system according to the manufacturer’s user guide. Statistical analysis was conducted using SPSS 16.0 software. Results: The study found that the plasma levels of total cholesterol (TC) (*P*=0.006), low-density lipoprotein cholesterol (LDL-C) (*P*=0.030), apolipoprotein A (ApoA) (*P*=0.038), and apolipoprotein B (ApoB) (*P*=0.022) distributed differently in patients with various alleles. Additionally, the AA genotype of rs731384 was found to be a protective factor against CAD in a recessive model (AA:AG+GG, odds ratio (OR) = 0.408, 95% confidence interval (95% CI) = 0.171–0.973, *P*=0.043). A significant association was found between the gene–environment interaction and CAD risk. The AA genotype along with high-density lipoprotein cholesterol (HDL-C) level ≥ 1.325 mmol/l significantly decreased the CAD risk (AA:AG+GG, OR = 0.117, 95% CI = 0.023–0.588, *P*=0.009). Conclusion: The mutant AA genotype of rs731384 seems to be a protective factor against CAD, and rs731384 plays an important role in the human metabolism of plasma lipids.

## Introduction

Cardiovascular diseases (CVDs), particularly coronary heart disease, lead to major human morbidities and mortalities worldwide [1]. In 2015, data indicated that 422.7 (95% confidence interval (95% CI: 415.53–427.87) million people were suffering from CVDs and 17.92 (95% CI: 17.59–18.28) million people died from coronary artery disease (CAD) [2]. The pathological foundation of CAD is generally considered to be coronary atherosclerosis, an inflammatory disorder caused by the formation of plaque and subsequent obstruction of the coronary arteries. However, the exact mechanism of this disease is still unclear. In recent years, genetic predisposition has been widely studied and was found to be closely associated with CAD [3,4].

miRNAs are short (20–24 nts) noncoding RNAs involved in the post-transcriptional regulation of gene expression in multicellular organisms by affecting both the stability and translation of mRNAs. The encoding gene of miR-130a is located in chromosome 11 and has a length of 89 nts. The sequence of miR-130a (Chr11: 57641198-57641286) is as follows: TGCTG CTGGC CAGAG CTCTT TTCAC ATTGT GCTAC TGTCT GCACC TGTCA CTAGC AGTGC AATGT TAAAA GGGCA TTGGC CGTGT AGTG. The mature products of miR-130a are segments with a length of 22 nts (miR-130a-3p and miR-130a-5p). Although only one exon is included, miR-130a has been identified to be significantly associated with familial hypercholesterolemia (FH), coronary atherosclerosis [5], CAD, and diabetes mellitus (DM) [6] in recent years.

In our previous studies, miR-221, miR-155, and miR-130a levels were found to be decreased in patients with CAD, and miR-130a may be an independent predictor for CAD [7]. Thus, in this study, a functional variant, rs731384, located in the promoter region of miR-130a, was selected to further investigate the molecular mechanism of CAD.

## Materials and methods

### Study subjects

To evaluate the genetic predisposition of CAD, 876 consecutive patients (641 males and 235 females) aged 32–84 years, who underwent coronary angiography for suspected or known coronary atherosclerosis at the Friendship Hospital of Ili Kazakh Autonomous Prefecture in China from 1 March 2010 to 31 April 2015 were recruited for the present study. The exclusion criteria of the study were as follows: subjects with spastic angina pectoris, infectious processes within 2 weeks, heart failure, adrenal dysfunction, and thyroid dysfunction. The diagnosis of CAD was according to the results of coronary angiography, which was performed by at least two experienced doctors simultaneously. Coronary arteries were cannulated using either the Judkins technique [8] or through a radial artery approach with 6F catheters. CAD subjects were defined as having at least one major epicardial vessel with >50% stenosis; control subjects were defined as having all of the major epicardial vessels with <50% stenosis [9]. The study was approved by the Ethics Committee of the First Affiliated Hospital of Nanjing Medical University and the Friendship Hospital of Ili Kazakh Autonomous Prefecture in China. All subjects provided written informed consents.

### Laboratory measurements

Four milliliters of venous blood was drawn after 12 h of fasting to perform biochemical assays on the second day of hospitalization. Laboratory measurements included the clinical parameters that have been reported to be associated with CAD [10–16]: total cholesterol (TC, mmol/l), triglyceride (TG, mmol/l), fasting blood glucose (FBG, mmol/l), creatine phosphokinase myoglobin isoenzyme (CKMB, U/l), fasting high-density lipoprotein cholesterol (HDL-C, mmol/l), fasting low-density lipoprotein cholesterol (LDL-C, mmol/l), apolipoprotein A (ApoA, g/l), and apolipoprotein B (ApoB, g/l) were determined by enzymatic procedures on an automated autoanalyzer (AU 2700 Olympus, 1st Chemical Ltd, Japan). The plasma preparation and RNA isolation were conducted according to previously reported protocols [17], and miR-130a was quantitated by RT-qPCR analysis [7].

### SNP selection

In our previous study, miR-130a was identified to be significantly associated with CAD [7]. The basic information, including the target gene sequence and gene loci (Chr11:57641198-57641286), of miR-130a was obtained from the NCBI website (www.ncbi.nlm.nih.gov). The Ensembl genome browser (www.ensembl.org) was used to screen the SNP sites in the promoter of miR-130a. The minor allele frequency (MAF) values were accessed on the website www.internationalgenome.org, and the SNP sites with MAF values <0.05 were excluded. The rs731384 SNP that resides in the promoter of miR-130a was selected in the study, and its MAF was 0.141 (>0.05). The primer design, PCR protocol, and the single base extension of the primers were designed using AssayDesigner 3.1 software (Sequenom Inc., San Diego, CA, U.S.A.). The primers were synthesized by a professional biotechnology company. All the primers were diluted according to the manufacturer’s user guide.

### Polymorphism genotyping

Genotypic polymorphisms were identified on the Agena MassARRAY system (Agena/Sequenom Inc., San Diego, CA, U.S.A.) according to manufacturer’s user guide.

The genomic DNA was extracted using a blood DNA extraction kit [Axygene Biotechnology (Hangzhou) Limited, Hangzhou City, China]. Quality control measures were conducted by 1.25% agarose gel electrophoresis (AGE), and the optical density (OD) values were detected using a Nanodrop 2000 spectrophotometer (Thermo, Wilmington, DE, U.S.A.). All the samples were stored at −20°C until use.

DNA samples were amplified via standard PCR. The primers of the target gene were designed using AssayDesigner 3.1 software (Sequenom Inc., San Diego, CA, U.S.A.) and were synthesized by a professional biotech company. Four microliters of PCR master mix was allocated into each well of 384-well plates and was mixed uniformly with 1 μl of template DNA (20 ng/μl). Microplate sealers were used to prevent evaporation during the reaction. The reaction environment and procedures were set as 94°C, 5 min; 94°C, 20 s; 56°C, 30 s; 72°C, 1 min; and 72°C, 3 min. A total of 45 cycles of repeating steps were conducted, and the completed reactions were stored at 4°C until use. The PCR products were treated with shrimp alkaline phosphatase (SAP) to remove the dNTPs. Two microliters of SAP mix and 5 μl of PCR products were mixed uniformly in each well of 384-well plates. Microplate sealers were used to prevent evaporation. The reaction environment and procedures were set as: 37°C, 20 min; 85°C, 5 min after centrifugation, and the products were stored at 4°C until use. Sequential single base extension (SBE) was then conducted. Two microliters of extending mix and 7 μl of PCR+SAP reaction reagent were mixed uniformly in each well of 384-well plates. Microplate sealers were used to prevent evaporation. The single base extension reaction was conducted after centrifugation. Resin purification was conducted, and the products were transferred into a 384-well spectroCHIP bioarray with a MassARRAY Nanodispenser RS 1000 (Agena, Inc). The genechip was analyzed with MALDI-TOF-MS (MassARRAY Analyzer 4.0, Agena, Inc). The original data and genotyping figures were obtained using MassARRAY TYPER4.0 software (Agena, Inc). The integrity and validity of the output were examined and submitted to the professional statisticians for further analysis.

### Statistical analysis

Statistical analysis was conducted using the Statistics Package for Social Sciences (ver. 16.0, SPSS Incorporated, Chicago, IL, U.S.A.). Skewed data are presented as the medians (interquartile ranges), normal data are presented as the means ± S.D. and categorical data are presented as the absolute values. The Chi-square tests, independent samples *t* tests, Mann–Whitney tests, Kruskal–Wallis tests, one-way ANOVA, and logistic regression analysis were conducted for data analysis. *P*<0.05 was considered significant in the two-tailed tests.

## Results

### Description of the study population

A total of 876 patients (641 males and 235 females) aged 32–84 years were recruited for the present study. The CAD cases (600) and controls (276) were distinguished by the results of coronary angiography. The plasma levels of CKMB and FBG were higher in the CAD group than in the control group, while the levels of HDL-C and miR-130a were significantly lower (*P*<0.05) in the CAD group than those in the control group (Table 1).

**Table 1:** Demographics of the study population

	CADs (*n*=600)	Controls (*n*=276)	*P*-value
Age (years)	61 (53-70)	58 (50-66)	**<0.001**
Gender (male/female)	465/135	176/100	**<0.001**
Smoking status (Yes/No)	286/314	111/165	**0.040**
Drinking status (Yes/No)	98/502	41/235	0.578
CKMB (U/l)	17 (13.40–23)	16 (13–20)	**0.001**
TC (mmol/l)	4.66 (3.88–5.51)	4.60 (3.91–5.33)	0.318
TG (mmol/l)	1.79 (1.24–2.48)	1.71 (1.17–2.39)	0.168
HDL-C (mmol/l)	1.32 (1.10–1.62)	1.40 (1.18–1.68)	**0.013**
LDL-C (mmol/l)	2.79 (2.20–3.48)	2.70 (2.14–3.36)	0.067
ApoA (g/l)	1.28 (1.14–1.45)	1.30 (1.18–1.49)	0.060
ApoB (g/l)	0.92 ± 0.23	0.90 ± 0.20	0.220
FBG (mmol/l)	5.21 (4.65–6.17)	4.87 (4.54–5.33)	**<0.001**
MiR-130a	2.98 (1.47–5.24)	3.56 (1.74–11.16)	**0.001**

Skewed data are presented as the medians (interquartile ranges), normal data are presented as the means ± S.D., and categorical data are presented as the absolute values. Smoking status, drinking status, and gender were examined by Chi-Square tests, the plasma level of ApoB was examined by Independent Samples *t* tests and the rest of the baseline characteristics were examined by Mann–Whitney tests. The value of miR-130a means the relative amount of miR-130a calculated by the 2^−Δ*c*_t_^method. Values in bold represent *P*-values of less than 0.05.

### Clinical parameter distributions in different genotypes and alleles

For further study, the subjects were divided into three groups according to different genotypes. AA represented the homozygote of minor alleles, AG represented the heterozygote, and GG represented the homozygote of major alleles. The plasma levels of TC (*P*=0.006), LDL-C (*P*=0.030), ApoA (*P*=0.038), and ApoB (*P*=0.022) distributed differently in various alleles (Table 2), suggesting that the SNP site rs731384 plays an important role in the human metabolism of plasma lipids. However, the miR-130a levels showed no differences between various alleles (*P*=0.652). The allele frequency in participants was consistent with Hardy–Weinberg equilibrium (HWE) (*P*=0.180).

**Table 2:** Clinical parameters distributed in different genotypes and alleles

	AA (*n*=21)	AG (*n*=264)	GG (*n*=591)	*P*-value
Age (years)	58 (49.5–71.5)	60 (51–68)	61 (52–69)	0.282
Gender (male/female)	17/4	199/65	425/166	0.411
Smoking status (Yes/No)	8/13	131/133	258/333	0.215
Drinking status (Yes/No)	3/18	43/221	93/498	0.960
CKMB (U/l)	16 (13–20)	16 (13–21)	17 (13.4–22)	0.114
TC (mmol/l)	4.52 (3.53–5.43)	4.42 (3.62–5.30)	4.72 (4.03–5.53)	**0.006**
TG (mmol/l)	1.92 (1.18–2.87)	1.71 (1.15–2.28)	1.78 (1.24–2.49)	0.197
HDL-C (mmol/l)	1.26 (1.04–1.50)	1.34 (1.13–1.60)	1.38 (1.14–1.68)	0.087
LDL-C (mmol/l)	2.43 (1.99–2.96)	2.65 (2.05–3.35)	2.80 (2.22–3.47)	**0.030**
ApoA (g/l)	1.23 (1.08–1.35)	1.30 (1.18–1.50)	1.29 (1.14–1.44)	**0.038**
ApoB (g/l)	0.92 ± 0.19	0.88 ± 0.24	0.93 ± 0.22	**0.022**
FBG (mmol/l)	5.2 (4.59–6.23)	5.08 (4.65–5.87)	5.08 (4.60–6.04)	0.987
MiR-130a	27.81 (26.53–28.74)	27.52 (26.65–28.24)	27.57 (26.63–28.62)	0.652
*P*-value for HWE				0.180

Skewed data are presented as the medians (interquartile ranges), normal data are presented as the means ± S.D., and categorical data are presented as the absolute values. The plasma level of ApoB was examined by one-way ANOVA, and the rest of the baseline characteristics were examined by Kruskal–Wallis tests. Abbreviation: MiR-130a, the relative amount of miR-130a calculated by the 2^−Δ*c*_t_^ method. Values in bold represent *P*-values of less than 0.05.

### Genotype and allele distributions in the case and control populations

The genotype distribution of rs731384 is shown in Table 3. The frequency of the AA genotype in the case group was significantly lower than that in the control group (*P*=0.039). However, the allele distribution in the case group was not in HWE (*P*=0.025). A logistic regression analysis was conducted to investigate the relationship between the genotypes of rs731384 and CAD risk. The polymorphism frequencies were consistent with HWE in the control group. As is shown in Table 4, the allele distributions in the study populations were consistent with a recessive model, and the AA genotype was a protective factor against CAD risk (AA:AG+GG, adjusted odds ratio (AOR) = 0.374, 95% CI = 0.154–0.906, *P=0.029).

**Table 3:** Distributions of genotypes and alleles in the case and control populations

Genotypes	CADs (*n*=600)	Controls (*n*=276)	*P*-value
GG	403 (67.2%)	188 (68.1%)	0.781
GA	187 (31.2%)	77 (27.9%)	0.327
AA	10 (1.7%)	11 (4.0%)	0.037
*P*-value for HWE	0.025	0.385	
Alleles	CADs (*n*=600)	Controls (*n*=276)	*P*-value
G	207 (17.25%)	99 (17.93%)	0.726
A	993 (82.75%)	453 (82.07%)	

AA, the homozygote of minor alleles; AG, the heterozygote; GG, the homozygote of major alleles. Genotypes and alleles are presented as the frequencies (%).

**Table 4:** Logistic analysis on the association between SNP rs731384 and CAD risk

Rs731384 (G>A)	CAD (*n*=600)	Control (*n*=276)	OR (95% CI)	*P*-value	AOR* (95% CI)	*P-value
GG	403	188	1.000 (reference)	0.097	1.000 (reference)	0.114
AG	187	77	0.424 (0.177–1.016)	0.054	0.392 (0.161–0.956)	0.039
AA	10	11	1.133 (0.825–1.555)	0.440	1.170 (0.845–1.622)	0.345
Dominant model (GG compared with GA+AA)	403/197	188/88	0.958 (0.706–1.299)	0.781	0.936 (0.684–1.282)	0.682
Recessive model (AA compared with GA+GG)	10/590	11/265	0.408 (0.171–0.973)	0.043	0.374 (0.154–0.906)	0.029

AA represents the homozygote of minor alleles, AG represents the heterozygote, GG represents the homozygote of major alleles. Abbreviations: OR, odds ratio; HWE, P-value for the HWE test.

* Adjusted for age, gender.

### The interactions of environmental and genetic factors in CAD prevalence

A receiver operating characteristic curve analysis was conducted to predict the prevalence of CAD. The areas under curve (AUCs) were 0.587 for age ≥ 59.5 years (95% CI: 0.547–0.628, *P*<0.001); 0.570 for CKMB ≥ 25.9 U/l (95% CI: 0.531–0.609, *P*=0.010); 0.552 for HDL-C < 1.325 mmol/l (95% CI: 0.512–0.592, *P*=0.013); 0.617 for FBG ≥ 5.445 mmol/l (95% CI: 0.579–0.656, *P*=<0.001); and 0.567 for the relative amount of miR-130a ≥ 6.521 (95% CI: 0.526–0.608, *P*=0.001) (Table 5). A crossover analysis was conducted to analyze the relationship between CAD risk and the interactions of environmental and genetic factors in a recessive model. As shown in Tables 6 and 7, the AA genotype was a protective factor for CAD in subjects with a status of HDL-C level ≥ 1.325 mmol/L (odds ratio (OR) = 0.117, 95% CI = 0.023–0.588, *P*=0.009, S = 1.105, AP = −0.718, AP* = 0.095, RERI = −0.084) compared with the reference population.

**Table 5:** Receiver operating characteristic curve analyses, including the optimal cut-off value and the Youden index for predicting CAD prevalence

Variables	AUC (95% CI)	*P*-value	Cut-off	Sensitivity	Specificity	Youden index
Age (years)	0.587 (0.547–0.628)	<0.001	59.5	0.572	0.562	0.134
Gender (male/female)	0.431 (0.390–0.473)	0.001	-	-	-	-
Smoking status (Yes/No)	0.538 (0.497–0.580)	0.070	-	-	-	-
Drinking status (Yes/No)	0.508 (0.466–0.549)	0.722	-	-	-	-
CKMB (U/l)	0.570 (0.531–0.609)	0.010	25.9	0.210	0.938	0.148
TC (mmol/l)	0.521 (0.481–0.561)	0.318	-	-	-	-
TG (mmol/l)	0.529 (0.487–0.570)	0.168	-	-	-	-
HDL-C (mmol/l)	0.552 (0.512–0.592)	0.013	1.325	0.609	0.503	0.112
LDL-C (mmol/l)	0.538 (0.498–0.579)	0.067	-	-	-	-
ApoA (g/l)	0.540 (0.499–0.580)	0.060	-	-	-	-
ApoB (g/l)	0.521 (0.480–0.561)	0.325	-	-	-	-
FBG (mmol/l)	0.617 (0.579–0.656)	<0.001	5.445	0.430	0.793	0.223
MiR-130a	0.567 (0.526–0.608)	0.001	6.521	0.304	0.817	0.121

The value of miR-130a means the relative amount calculated by the 2^−Δ*c*^_t_ method. AUC, the closer the value is to 0.5, the less predictive it is.

**Table 6:** CAD incidence by interactions with environmental factors such as age, gender, smoking status, drinking status, the plasma level of CKMB, HDL-C, FBG, and miR-130a

Characteristics	Recessive model of rs731384			
	GG+AG		AA	
Age (years)	OR (95% CI)	*P*-value	OR (95% CI)	*P-*value
<59.5	1.000 (Reference)	0.000	0.840 (0.262–2.694)	0.769
≥59.5	1.774 (1.324–2.377)	0.000	0.300 (0.074–1.217)	0.092
Gender				
Male	1.000 (Reference)	0.000	0.414 (0.157–1.092)	0.075
Female	0.509 (0.371–0.698)	0.000	0.123 (0.013–1.189)	0.070
CKMB (U/l)				
<25.9	1.000 (Reference)	0.000	0.537 (0.220–1.307)	0.170
≥25.9	4.226 (2.457–7.270)	0.000	-	-
HDL-C (mmol/l)				
<1.325	1.000 (Reference)	0.002	0.561 (0.179–1.752)	0.320
≥1.325	0.640 (0.477–0.860)	0.003	**0.117 (0.023–0.588)**	**0.009**
FBG (mmol/l)				
<5.445	1.000 (Reference)	0.000	0.346 (0.114–1.047)	0.060
≥5.445	2.866 (2.043–4.022)	0.000	1.558 (0.300–8.102)	0.598
miR-130a				
<6.521	1.000 (Reference)	0.000	0.439 (0.157–1.227)	0.116
≥6.521	0.518 (0.371–0.724)	0.000	0.192 (0.035–1.057)	0.058

miR-130a, the relative amount calculated by the 2^−Δ*c*^_t_ method. Values in bold represent *P*-values of less than 0.05.

**Table 7:** The indexes of the synergistic effects between the recessive model of rs731384 and risk factors

Variables	S	AP	AP*	RERI
HDL-C	1.105	−0.718	0.095	−0.084

Abbreviations: AP, attributable proportion of interaction; AP*, the attributable proportion of interaction caused only by gene and environment; RERI, relative excess risk of interaction; S, Rothman’s synergy index for an interaction.

## Conclusion

In the present study, the results revealed that rs731384 plays an important role in the human metabolism of plasma lipids. The AA genotype was a protective factor against CAD compared with the AG+GG genotypes (AOR = 0.374, 95% CI = 0.154–0.906, *P=0.029), and the protective effect was significantly enhanced when the AA genotype was present with an HDL-C level ≥1.325 mmol/l (OR = 0.117, 95% CI = 0.023–0.588, *P*=0.009). The results suggested that both environmental and genetic factors work together in the occurrence and development of CAD.

## Discussion

The association between miR-130a and CAD has been widely investigated. In previous studies, miR-130a was found to be an important angiogenic miRNA, and its dysregulation might contribute to endothelial progenitor cell (EPC) dysfunction in CAD patients [18,19]. Endothelial cells comprise the continuous monolayer of cells covering the inner surfaces of vessels and have significant biological functions, including regulation of thrombosis and coagulation, dilation of vascular smooth muscle, suppression of platelet adhesion and aggregation [20,21], secretion of vasoactive substances and regulation of angiostasis [22]. Studies have found that normal endothelial cells help prevent lipid metabolism disorders, which are the leading risk factors of CAD. The low-density lipoprotein permeability of endothelial cells increases abnormally in hyperlipidemia subjects, which contributes to vessel intima lipidosis and the development of CAD [23]. Additionally, miR-130a was found to alleviate human coronary artery endothelial cell injury, inflammatory responses, cardiac dysfunction, and myocardial infarction by down-regulating PTEN and activating the PI3K/Akt/eNOS signaling pathway [24,25]. Last year, an hsa-miR-130a-3p-mediated circRNA–mRNA ceRNA network showed that nine circRNAs promote transient receptor potential cation channel subfamily M member 3 (TRPM3) expression by inhibiting hsa-miR-130a-3p in CAD patients [26].

In our previous study, it was shown that miR-130a was decreased in patients with CAD and may be an independent predictor of CAD. To further investigate the genetic mechanism by which miR-130a was distributed differently between CAD patients and non-CAD subjects, we selected the SNP site rs731384, located in the promoter of miR-130a, and a case–control study was conducted.

In the present study, the AA genotype was a protective factor against CAD compared with the AG+GG genotype, and the protective effect was significantly enhanced when the AA genotype was present with an HDL-C level ≥ 1.325 mmol/l. Furthermore, direct evidence from the present study showed that SNP rs731384 plays a significant role in determining the plasma lipid levels in CAD patients. The plasma levels of TC, LDL-C, ApoA, and ApoB were determined to be different due to alleles.

SNP function prediction in the National Institutes of Health database showed that a transcription factor binding site YY1-Q6 (core match score = 1, matrix match score = 0.989, sequence: GCCATtttc) is highly likely to be a potential target of rs731384. YY1, also called Yin-Yang1, is a ubiquitous transcription factor with abundant cellular functions. Animal experiments have shown that YY1 promotes liver steatosis and lipotoxicity by the suppression of Farnesoid X receptorin (FXR) [27,28]. FXR plays a key role in maintaining lipid metabolic homeostasis, regeneration of liver cells and prevention of liver fibrosis, while YY1 regulates lipid metabolism via the FXR-SHP signaling axis by targetting intron 1 of the *FXR* gene [29].

**Figure 1:**
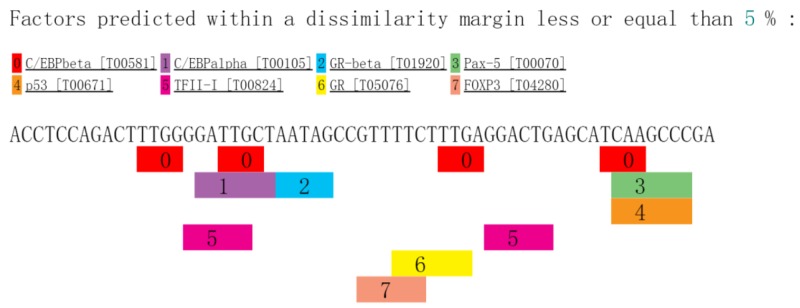
Transcription factor prediction by the PROMO database

Additionally, another transcription factor prediction by the PROMO database showed that Forkhead Box P3 (FOXP3) is also a possible target of rs731384 (Figure 1). FOXP3 is closely related to the function of regulatory T cells (Tregs), and reduced expression of FOXP3 and decreased Treg levels have been observed in CAD patients in recent studies [30–33]. A study implied that the FOXP3 gene may exert an influence on immune responses and result in unstable plaques in CAD patients [34].

We hypothesize that the SNP rs731384 may have the potential to reduce the occurrence and development of CAD by regulating human lipid metabolism via binding the YY1 transcription factor. However, an individual SNP site is not strong enough to make a difference between CAD patients and healthy people. More studies covering novel SNP sites need to be conducted. Our study first investigated the relationship between rs731384 and CAD and the role rs731384 plays in lipid metabolism amongst Chinese populations. It confirms a previous study that found that lipometabolism regulated by gene expression may be a potential risk factor and predictor of CAD [35,36].

## Limitations

Several limitations existed in the present study. First, the sample size of subjects with the AA genotype was small, and the genotype distribution was not in HWE in the case group. More specimens are needed for further investigation. Second, only one SNP site was focussed on in the present study, meaning that the range of study was relatively narrow. Third, the absence of further functional validation made the results less convincing.

## Abbreviations

ApoA: apolipoprotein A
ApoB: apolipoprotein B
AOR: adjusted odds ratio
AUC: area under curve
CAD: coronary artery disease
CeRNA: competing endogenous ribonucleic acid
CircRNA: circular ribonucleic acid
CKMB: creatine phosphokinase myoglobin isoenzyme
CVD: cardiovascular disease
FBG: fasting blood glucose
FOXP3: Forkhead Box P3
FXR: Farnesoid X receptorin
HDL-C: high-density lipoprotein cholesterol
HWE: Hardy–Weinberg equilibrium
LDL-C: low-density lipoprotein cholesterol
MAF: minor allele frequency
OR: odds ratio
PTEN: phosphatase and tensin homolog deleted on chromosome ten
RT-qPCR: reverse transcription-quantitative polymerase chain reaction
SAP: shrimp alkaline phosphatase
SHP: small heterodimer partner
SNP: single nucleotide polymorphism
TC: total cholesterol
TG: triglyceride
TRPM3: transient receptor potential melastatin 3
95% CI: 95% confidence interval
